# Pathogenic Mitochondrial DNA Mutation Load Inversely Correlates with Malignant Features in Familial Oncocytic Parathyroid Tumors Associated with Hyperparathyroidism-Jaw Tumor Syndrome

**DOI:** 10.3390/cells10112920

**Published:** 2021-10-28

**Authors:** Monica De Luise, Luisa Iommarini, Lorena Marchio, Greta Tedesco, Camelia Alexandra Coadă, Andrea Repaci, Daniela Turchetti, Maria Lucia Tardio, Nunzio Salfi, Uberto Pagotto, Ivana Kurelac, Anna Maria Porcelli, Giuseppe Gasparre

**Affiliations:** 1 Department of Medical and Surgical Sciences (DIMEC), University of Bologna, 40138 Bologna, Italy; monica.deluise2@unibo.it (M.D.L.); lorena.marchio2@unibo.it (L.M.); greta.tedesco@studio.unibo.it (G.T.); camelia.coada@unibo.it (C.A.C.); daniela.turchetti@unibo.it (D.T.); uberto.pagotto@unibo.it (U.P.); ivana.kurelac@unibo.it (I.K.); 2 Center for Applied Biomedical Research (CRBA), University of Bologna, 40138 Bologna, Italy; luisa.iommarini2@unibo.it (L.I.); annamaria.porcelli@unibo.it (A.M.P.); 3 Department of Pharmacy and Biotechnology (FABIT), University of Bologna, 40126 Bologna, Italy; 4 Division of Endocrinology and Diabetes Prevention and Care, IRCCS Azienda Ospedaliero-Universitaria di Bologna, 40138 Bologna, Italy; andrea.repaci@aosp.bo.it; 5 Division of Medical Genetics, IRCSS Azienda Ospedaliero-Universitaria di Bologna, 40138 Bologna, Italy; 6 Unit of Pathology, IRCCS S.Orsola University Hospital, 40138 Bologna, Italy; marialucia.tardio@aosp.bo.it; 7 Pathology Unit, IRCCS Giannina Gaslini Children’s Research Hospital, 16147 Genova, Italy; nunziosalfi@gaslini.org; 8 Interdepartmental Center of Industrial Research (CIRI) Life Science and Health Technologies, University of Bologna, 40064 Ozzano dell’Emilia, Italy

**Keywords:** mitochondrial DNA mutations, familial oncocytic tumors, respiratory complexes, hyperparathyroidism-jaw tumor syndrome, parathyroid cancer

## Abstract

While somatic disruptive mitochondrial DNA (mtDNA) mutations that severely affect the respiratory chain are counter-selected in most human neoplasms, they are the genetic hallmark of indolent oncocytomas, where they appear to contribute to reduce tumorigenic potential. A correlation between mtDNA mutation type and load, and the clinical outcome of a tumor, corroborated by functional studies, is currently lacking. Recurrent familial oncocytomas are extremely rare entities, and they offer the chance to investigate the determinants of oncocytic transformation and the role of both germline and somatic mtDNA mutations in cancer. We here report the first family with Hyperparathyroidism-Jaw Tumor (HPT-JT) syndrome showing the inherited predisposition of four individuals to develop parathyroid oncocytic tumors. MtDNA sequencing revealed a rare ribosomal RNA mutation in the germline of all HPT-JT affected individuals whose pathogenicity was functionally evaluated via cybridization technique, and which was counter-selected in the most aggressive infiltrating carcinoma, but positively selected in adenomas. In all tumors different somatic mutations accumulated on this genetic background, with an inverse clear-cut correlation between the load of pathogenic mtDNA mutations and the indolent behavior of neoplasms, highlighting the importance of the former both as modifiers of cancer fate and as prognostic markers.

## 1. Introduction

Somatic mitochondrial DNA (mtDNA) mutations have been reported in almost all cancer types at variable degrees of mutation load [1,2,3,4,5], although their role as modifiers during cancer initiation or progression is still to be elucidated. Notwithstanding this, it is clear that variants that severely affect the respiratory chain are usually maintained at low heteroplasmy, while those that achieve homoplasmy have been found to be neutral [1].

Despite being nowadays a dogma of cancer biology that a functional mitochondrial oxidative phosphorylation (OXPHOS) be essential to sustain tumor progression [6,7,8], in some cases pathogenic mtDNA mutations affecting mitochondrial function have been reported to shift towards homoplasmy in association with rare benign oncocytic tumors (oncocytomas), escaping purifying selection [9]. Overall, homoplasmic disruptive mutations, such as those affecting respiratory complex I (CI) structural integrity and function, have been long considered hallmarks of oncocytic transformation and are responsible for reduced tumorigenic potential and a less aggressive behavior. This indolent phenotype is associated with an impaired metabolism as well as the inability of cancer cells to promptly adapt to hypoxia [10,11,12,13].

Oncocytomas are epithelia-derived human neoplasms characterized by an aberrant accumulation of swollen and deranged mitochondria within the cytoplasm. These tumors, found in endocrine and exocrine tissues [14], mainly occur in sporadic forms, albeit rare familial cases have been reported within the phenotypic spectrum of Birt-Hogg-Dubè (BHD; OMIM:135150) [15,16] or Cowden syndrome (CS, OMIM:158350) [17,18], and of Familial Non-medullary Thyroid Carcinoma (OMIM: 188470) [19,20]. Even though oncogenic drivers are known in these syndromes, i.e., the Folliculin (*FLCN*) gene for BHD and Phosphatase and Tensin Homolog (*PTEN*) for CS, the search for a role of such nuclear genes in triggering oncocytic transformation has failed to date, and it is likely therefore that other modifiers impinge on the phenotypic change that occurs in a few, but not all syndromic individuals. Indeed, oncocytic change appears to be a secondary event during cancer progression, with mtDNA mutations potentially playing a role in contributing to generate a retrograde organelle-nucleus signaling towards a mitochondrial biogenesis increase to compensate the respiratory damage [21].

In the present study, we report a family affected by the autosomal dominant hereditary disease Hyperparathyroidism-Jaw Tumor (HPT-JT) syndrome (OMIM:145001) with a predisposition to develop oncocytic neoplasms in the parathyroid glands. To the best of our knowledge, this is the first time that familial oncocytic tumors are reported within the syndromic spectrum of HPT-JT. The latter is caused by germline mutations of the tumor suppressor *CDC73* [22] and somatic LOH of this gene has been associated with parathyroid carcinomas, though never with an oncocytic phenotype [23,24].

As disruptive mtDNA lesions are genetic hallmarks of oncocytic transformation, we searched for mtDNA mutations and revealed a rare pathogenic ribosomal RNA change in the germline of all HPT-JT affected individuals, an unusual genetic lesion on which random somatic mutations built up in cancer cells. We finally show an inverse clear-cut correlation between the load of pathogenic mtDNA mutations and the indolent behavior of neoplasms, highlighting the importance of the former in modifying cancer fate.

## 2. Materials and Methods

### 2.1. Samples

**Figure 1:**
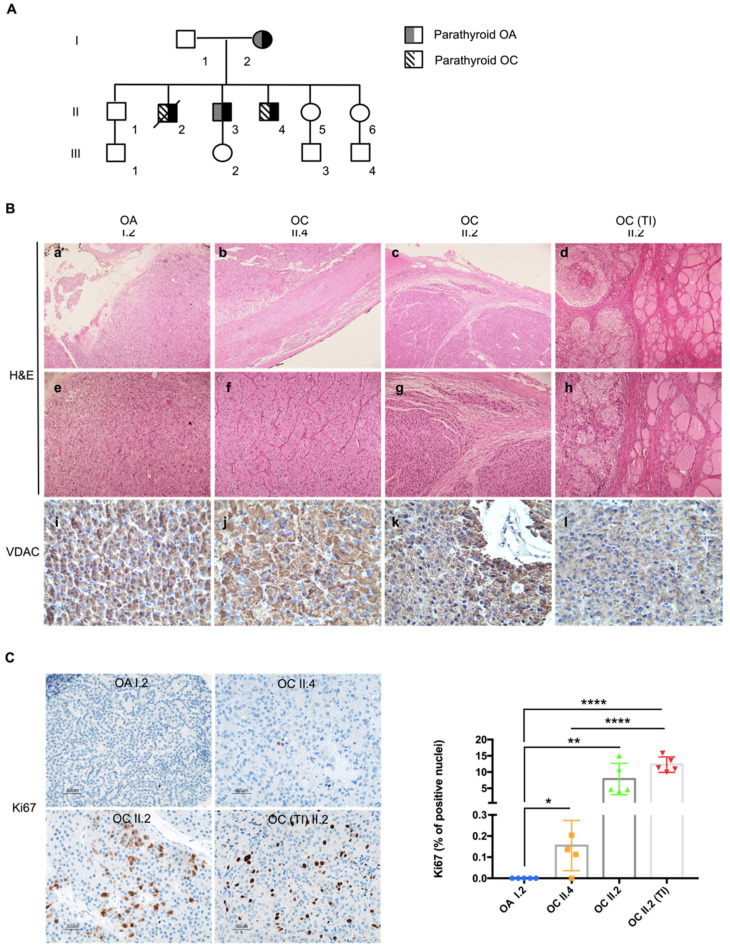
HPT-JT family shows a predisposition to develop parathyroid tumors characterized by an oncocytic phenotype. (**A**) Pedigree of the family with HPT-JT. In black: family members affected by the syndrome. (**B**) Histological analysis of the tumors. Haematoxylin and eosin (H&E) staining (**a**–**h**); magnification: 5× in **a**–**d**; 10× in (**e**–**h**). Immunohistochemistry analysis of FFPE sections from HPT-JT tumors, using antibody against VDAC (**i**–**l**); magnification 40×). OA—Parathyroid oncocytic adenoma, OC—parathyroid oncocytic carcinoma, TI—thyroid infiltration of the OC. (**C**) Representative images of Ki67 immunohistochemistry staining (magnification 20×) and quantification of cells displaying Ki67 positive nuclei in HPT-JT tumors. Scale bar 50 µm. Data are mean ± SD. *p*-value < 0.05 (*); *p*-value < 0.01(**); *p*-value < 0.0001 (****).

Samples were obtained from the pathology unit of Bologna University Medical School at S. Orsola-Malpighi Hospital. Blood was collected from individuals I.2, II.3 and II.4 of a family carrying a large germline heterozygous deletion of the gene *CDC73* and, therefore, clinically diagnosed as affected by HPT-JT syndrome (Figure 1A). Formaldehyde Fixed-Paraffin Embedded (FFPE) tissue sections were available from four individuals of the family: two parathyroid oncocytic adenomas (I.2 and II.3); parathyroid oncocytic carcinoma (II.2), thyroid infiltration of the parathyroid oncocytic carcinoma and the respective normal thyroid tissue (II.2); parathyroid oncocytic carcinoma and respective normal parathyroid tissue (II.4). Written informed consent was given by all individuals analyzed (protocol N.497/2018/Sper/AOUBo).

### 2.2. Mitochondrial DNA Sequencing and Analysis

Oncocytic tumors were manually macro-dissected from FFPE sections, avoiding contamination with the adjacent normal tissue. DNA from blood samples was extracted using GenElute™ Mammalian Genomic DNA Miniprep Kit (SIGMA) whereas FFPE-derived DNA was extracted using the QIAamp DNA FFPE Tissue Kit (QIAGEN). The entire sequence of the mitochondrial genome was obtained and analyzed as previously described [25], using the revised Cambridge Reference Sequence (rCRS) of the human mitochondrial DNA as a reference (Ref.Seq. NC_012920.1). Briefly, 2–5 ng/sample of genomic DNA were amplified with the MitoALL Resequencing kit [25]. PCR products were purified with Multiscreen plates for DNA clean-up (Millipore, #MSNU03050). Direct sequencing of the PCR product was performed with BigDye™ Terminator v1.1 Cycle Sequencing Kit (Thermo Scientific, Waltham, MA, USA). Sequences were run in the ABI 3730 DNA Analyzer for Sanger sequencing. Electropherograms were analyzed with SeqScape^®^ software (Applied Biosystems, Waltham, MA, USA). MtDNA variants were defined as somatic by comparing the subjects’ blood and/or the adjacent normal tissue with tumor tissue.

The prioritization of mitochondrial variants was carried out using MToolBox collection of annotations [26]; Human Mitochondrial Database (http://www.hmtdb.uniba.itHmtDB, accessed on 15 September 2021) [27,28], MITOMAP (http://www.mitomap.org/MITOMAP, accessed on 15 September 2021) [29], ClinVar [30] and their frequency in individuals involved in 1000 Genomes Project. Pathogenicity potential was assessed using the criteria described in the latest version of the ACMG/AMP standards and guidelines for mtDNA variant interpretation [31,32]. Allele frequency was also sourced from Helix mitochondrial variant database, HelixMTdb. Accessed at Helix.com/MITO on 15 September 2021.

PolyPhen [33,34] (www.tux.embl-heidelberg.de/ramensky/polyphen.cgi, accessed on 15 September 2021), was used to predict the possible impact of amino acid substitutions on the corresponding proteins. For rRNA variants, evolutionary conservation was evaluated by multiple sequence alignment using SEA view, an interface for molecular phylogeny [35]. Prediction of 16S conservation was performed using the protein multiple sequence alignment program MUSCLE (MUltiple Sequence Comparison by Log- Expectation) and the NCBI Reference Sequences of the complete mitochondrial genome for all species analyzed.

Protein sequences were obtained using UniProtKB (http://www.uniprot.org, accessed on 15 September 2021): Cytochrome *b* ID: P00156; ND1 ID: P03886; ND6 ID: P03923. Amino acid changes induced by missense mtDNA variants were mapped on their respective protein tertiary structures by using UCSF Chimera software version 1.15 [36]. Human crystal structures of the mitochondrial respiratory chain CI and complex III (CIII) were downloaded from RCSB—The Protein Data Bank (http://www.rcsb.org/, accessed on 15 September 2021) using the “Fetch by ID” function (CI—5XTD, CIII—5XTE).

### 2.3. Heteroplasmy Assessment and Allele Separation Analysis of MT-RNR2 Variants

Heteroplasmic levels of the m.2356A>G/*MT-RNR2* were evaluated by denaturing High-Performance Liquid Chromatography (dHPLC) in tumor and healthy tissues of HPT-JT family members. Specific primers (forward 5′-AAGCTCAACACCCACTACCT, reverse 5′-GCGGTGCCTCTAATACTGGT141) were used as previously described [37].

To evaluate whether the m.2356A>G/*MT-RNR2* and m.2635G>A/*MT-RNR2* were on the same molecule, the fragment of mtDNA including both variants was amplified, and PCR products were cloned and a total of 50 white colonies were screened and analyzed as previously described [37].

### 2.4. Histological Analyses

Haematoxylin-Eosin (H&E) and immunohistochemical (IHC) analysis was performed on 4 µm-thick FFPE serial sections. Due to the scarcity of II.3 tumor sample only H&E staining was performed on FFPE sections. Sections were processed as previously described [38]. The Envision Detection System (Dako, #K4007 and #K4011) was used for blocking, primary/secondary antibody incubations and DAB staining, according to the manufacturer’s instructions. The following primary antibodies were applied: mouse monoclonal anti-Ki-67 (1:100, Dako #M7240); rabbit polyclonal anti-VDAC (1:1000, Abcam #ab34726); mouse monoclonal anti-NDUFS4 (1:1000, Abcam #55540); mouse monoclonal anti-MTCOI (1:1000, Abcam #ab14705). Cells with Ki-67 positive nuclei were counted at 20x magnification in 5 fields of view per tumor. White balance for microscopy images was carried out in Photoshop CC 2021, using the same preset settings for all images. NDUFS4 and COX-I expression levels are represented as quantitative score (QS), calculated as the product of the percentage of positive cells (P) and the staining intensity (I), as previously reported [38]. The final QS derives from a blind test of four independent operators and is a value within the range of 0 (negative staining) and 12 (strong positive staining).

### 2.5. Cybrids Generation

Platelets from individual II.3 carrying the homoplasmic m.2356G>A/*MT-RNR2* variant were used as mitochondrial donors to obtain homoplasmic mutant cybrids, while those from individual II.4 bearing the same variant in heteroplasmy allowed us to generate the wild-type clones. Human osteosarcoma 143B.TK^−^ cells depleted for their mtDNA (Rho0 cells) were used as recipients [39]. After cell fusion and cybrid selection, multiple clones were screened to determine the mutation load as described above. Subsequently, pools of wild-type and homoplasmic mutant clones were generated. Cells were grown in Dulbecco’s modified Eagle medium (DMEM high glucose) supplemented with 10% fetal calf serum, 1 mM L-glutamine, 100 U/mL penicillin and 100 μg/mL streptomycin, in an incubator with a humidified atmosphere of 5% CO_2_ at 37 °C.

### 2.6. Complex I Re-Assembly Kinetics Assay

To follow the assembly kinetics of CI, cells were incubated for 10 days with culture medium containing 20 μg/mL doxycycline, a reversible inhibitor of mitochondrial translation [40]. Subsequently, the cells were cultured in standard medium and harvested after 8, 16, 24 and 48 h to follow the recovery of CI assembly, indicative of mtDNA-encoded CI subunits translation kinetics and thus used for the indirect evaluation of the efficiency of mitochondrial protein synthesis. Mitochondrial-enriched fractions were obtained by digitonin treatment [41]. Briefly, 5–8 × 10^6^ cells were harvested by trypsinization, washed twice in cold PBS and incubated in ice for 10 min with 2 mg/mL digitonin (Calbiochem, #3000410). Next, cold PBS was added and a centrifugation at 10,000× *g* for 10 min and 4 °C was performed. Mitochondrial pellets were suspended in mitochondrial buffer (750 mM aminocaproic acid, 50 mM Bis-Tris, pH = 7) and solubilized by adding n-dodecyl β-d-maltoside (DDM) (Thermo Scientific, #89903) with a DDM/protein ratio of 2.5 g/g. Suspension was incubated on ice for 10 min and then centrifuged at 13,000× *g* for 15 min. Aliquots of supernatants (80 µg protein) were separated by 4–16% high resolution Clear Native Electrophoresis (hrCNE) followed by CI In-Gel Activity (CI-IGA) assay [42], which allowed to follow the time-course of CI assembly. Densitometric analysis was performed using ImageJ [43].

### 2.7. Statistical Analysis

Statistical analyses were performed using GraphPad Prism v.8 (GraphPad Software Inc., San Diego, CA, USA). A two-tailed unpaired Student’s *t*-tests assuming equal variances was performed. Data were expressed as mean ± SD. Statistical significance was defined by *p*-value < 0.05.

## 3. Results

### 3.1. Patients with HPT-JT Syndrome Develop Tumors with Oncocytic Phenotype

A family with HPT-JT syndrome, previously described [44], was brought to our attention due to the peculiar occurrence of oncocytic phenotype in the lesions of the affected individuals. The family had been referred to for a previous history of parathyroid carcinoma and adenoma, and members had presented with hyperparathyroidism, hypercalcemia and nephrocalcinosis. Genetic analysis had revealed a deletion of the first 10 exons of the *CDC73* gene with the following breakpoints: left193,083,733–193,083,949 and right 193,126,404–193,126,441 (GRCh37/hg19), confirmed by MLPA analysis and array CGH [44]. Subject I.2 was diagnosed with an oncocytic parathyroid adenoma at the age of 62; II.2 developed an oncocytic parathyroid carcinoma with a thyroid infiltration at the age of 42 and died at 46; II.3 developed an oncocytic parathyroid adenoma at the age of 47 and remained disease-free to date; II.4 developed an oncocytic parathyroid carcinoma at the age of 37 and remained disease-free to date (Figure 1A). Haematoxylin/eosin staining of FFPE sections revealed an intense eosinophilic cytoplasm confirming the oncocytic nature of the lesions developed by I.2, II.4, II.2 and the thyroid infiltration of II.2 (Figure 1B(a–h) and Supplementary Figure S1). The oncocytic adenoma of subject I.2 lacked a thick capsule, vascular invasion or invasion to adjacent tissues, therefore it was classified as oxyphilic adenoma of the parathyroid (Figure 1B(a,e)). The two carcinomas were characterized by an expansive growth, well-defined round borders of the capsule and by the growth of dense fibrous tissue at the edge of the tumor, indicative of a desmoplastic reaction causing thickening of the capsule (Figure 1B(b,c)), a process usually associated with malignancy. The tumor of II.2 showed an infiltrative growth of the neoplastic tissue through the collagenous fibers of the capsule along within an altered tissue architecture (Figure 1B(c,g)), both distinctive features of malignancy. Invasion was evident in the adjacent normal thyroid (Figure 1B(d–h)), in association with a higher Ki67 proliferative index (7.9% in tumor of II.2 and 12.3% in the infiltrative part of the same mass), compared to I.2 (0%) and II.4 (0.2%) (Figure 1C). Taken together, members of this family affected with HPT-JT developed neoplasms characterized by a distinct oncocytic phenotype, which was confirmed by a strong positive immunostaining for the mitochondrial VDAC protein (Figure 1B(i–l)).

### 3.2. Oncocytic Tumors Associated with HPT-JT Syndrome Accumulate Different Somatic mtDNA Mutations

Oncocytic neoplasms have been significantly associated with occurrence of damaging mtDNA mutations, both somatic and germline, regardless of the site where they arise [45,46]. Nonetheless, despite being frequent genetic lesions in sporadic oncocytomas, including the parathyroids [47], only rarely such mutations were found in familial cases [48]. In order to understand whether mtDNA mutations were associated to the oncocytic phenotype in this family, sequencing of the entire mtDNA was performed. Analysis of the tumor-extracted mtDNA revealed common polymorphisms defining the mitochondrial macrohaplogroup H in all samples (Supplementary Table S1). Additionally, each tumor was shown to harbor a different number of mtDNA mutations that were mostly not shared among the family members (Table 1).

**Figure 2:**
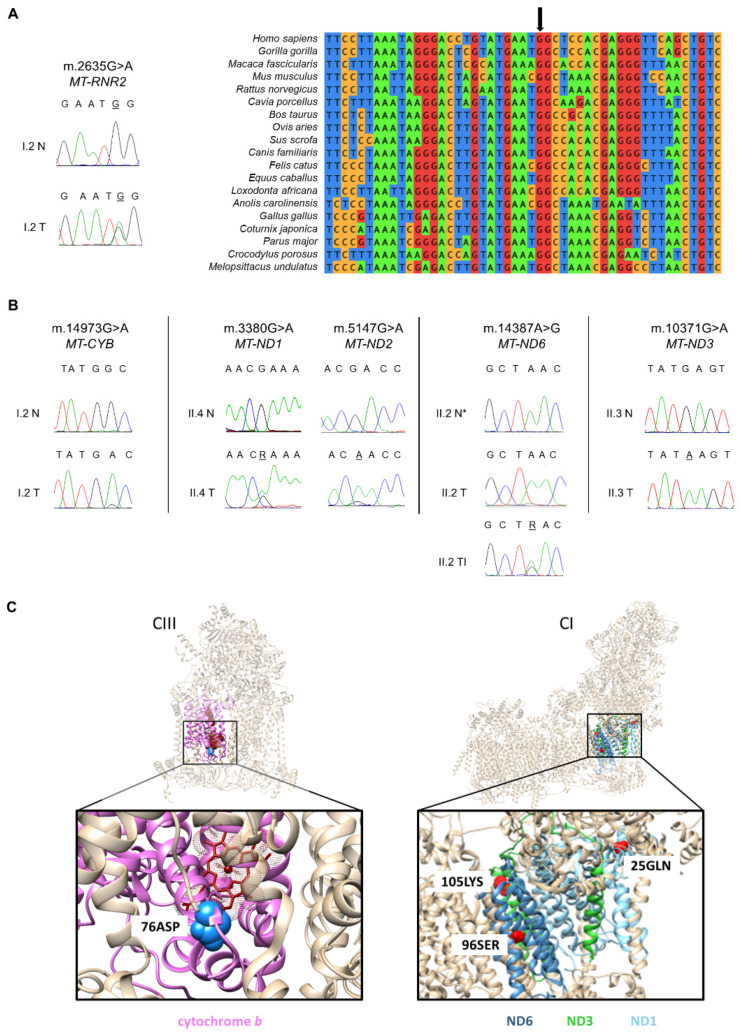
Oncocytic tumors of HPT-JT patients accumulate different somatic mtDNA mutations. (**A**) Electropherograms and phylogenetic conservation analysis of the somatic m.2635G>A in the *MT-RNR2* gene found in the tumor (T) from I.2. Blood DNA was used as the control normal tissue (N). The black arrow indicates the position of the highly conserved guanine. (**B**) Electropherograms showing the somatic mtDNA mutations found in HPT-JT tumors (T) and the respective sequences of the blood DNA (N) or adjacent normal tissue (N*), which were used as controls to identify a potential germinal origin. (**C**) Mapping of the pathogenic amino acid changes caused by the mtDNA mutations in genes encoding subunits of Complex III (CIII) and Complex I (CI) in HPT-JT patients. Cytochrome *b* is colored pink, the amino acid change G76D is labeled as 76ASP. ND1 helices are colored light blue and the amino acid change R25Q is labeled as 25GLN. ND6 is colored cyan and the amino acid change L96S is labeled as 96SER. ND3 is colored green and the amino acid change E105K is labeled as 105LYS. MtDNA encoded subunits affected are shown in colors while the rest of the subunits are in tan; affected amino acids are indicated in blue for CIII and in red for CI.

Comparison with the constitutive mtDNA sequence revealed all these individual mutations but one to be tumor-specific. We first focused on the somatic variants characterization. In detail, the I.2 adenoma harbored the very rare heteroplasmic m.2635G>A in *MT-RNR2* (Figure 2A, Supplementary Table S1). Despite several bioinformatics tools are conceived to predict RNA secondary structures, it is difficult to assign the pathogenic potential of mitochondrial rRNA variants. To date, except for the association of mtDNA mutations affecting ribosomal encoding genes with known pathologies, only a few methods were suggested as predictors of pathogenicity [48]. Since no PhyloP conservation score [49] was available for the m.2635G>A variant (Supplementary Table S1), we performed a multiple species sequence alignment of *MT-RNR2* orthologues. We showed that the m.2635G>A affects a highly conserved position of the gene across 19 vertebrates (Figure 2A), suggesting its potential pathogenicity. Individual I.2 also harbored the somatic m.14973G>A/*MT-CYB* mutation in respiratory Complex III (CIII; Figure 2B), previously associated with a clear cell renal cell carcinoma (CCRCC), as annotated in COSMIC (Catalog of Somatic Mutations in Cancer; [50], Mutation ID: COSM1138288). The m.14973G>A mutation causes the substitution at position 76 of the small non-polar amino acid glycine with the sterically bulky and negatively charged aspartic acid and was predicted as pathogenic (Table 1, Supplementary Table S1). Since the m.14973G>A affects a loop in the secondary structure located near the heme b_L_ in the Qo-site of cytochrome *b* (Figure 2C), it is reasonable to hypothesize that the variant may cause conformational changes likely influencing electron transport. In the II.4 parathyroid carcinoma we detected the heteroplasmic m.3380G>A, p.R25Q variant (Figure 2B,C) in the *MT-ND1* gene (Table 1), a hotspot for mutations associated to oncocytic tumors [51]. Interestingly, the mutation is causative of Mitochondrial Encephalomyopathy, Lactic Acidosis, and Stroke-like episodes (MELAS) [52] and therefore pathogenic. Additionally, the silent somatic m.5147G>A in *MT-ND2* was found in the II.4 tumor tissue (Table 1 and Figure 2B), which is highly unlikely to be pathogenic despite its high heteroplasmy.

Sequencing of mtDNA in the tumors developed by patient II.2 revealed the presence of the somatic transition m.14387A>G/*MT-ND6* within the thyroid infiltration of the parathyroid carcinoma (Figure 2B). The m.14387A>G was heteroplasmic in the parathyroid tumor (Figure 2B) and absent in the normal thyroid. This change lies within the transmembrane helix IV of ND6 subunit and causes a substitution of the non-polar amino acid leucine at position 96 with a polar serine (Figure 2C, [53]). The variant had a high pathogenicity score according to HmtVar (Table 1, Supplementary Table S1), which however was present with a low heteroplasmic load and therefore unlikely to determine a phenotypic effect.

Finally, the sequencing analysis of the parathyroid oncocytoma from patient II.3 revealed the homoplasmic somatic m.10371G>A in the CI *MT-ND3* gene (Figure 2B). The variant was predicted to be pathogenic (Table 1, Supplementary Table S1) because of the substitution of a glutamic acid at position 105 with a lysine, that replaces the negative charge with a positive one (Figure 2C).

### 3.3. A Rare Pathogenic Germline mtDNA Mutation Shifts Bidirectionally in Correlation with the Degree of Malignancy

**Figure 3:**
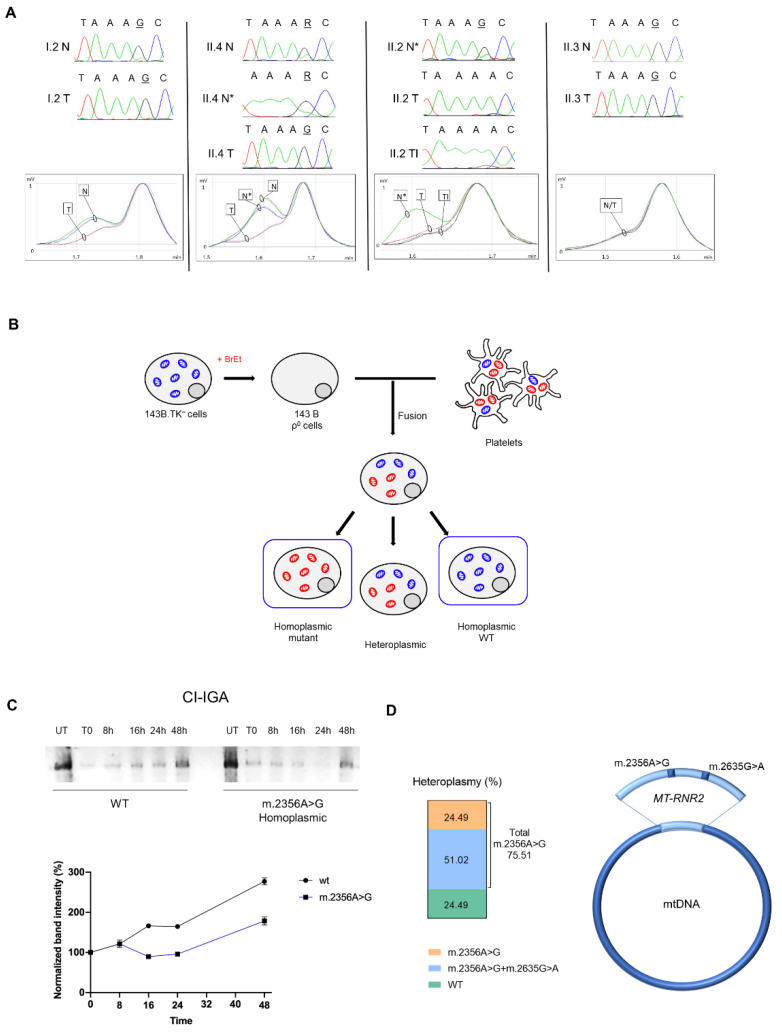
The homoplasmic shift of the m.2356A>G affects mitoribosome activity. (**A**) Electropherograms and dHPLC analysis of the germline m.2356A>G in *MT-RNR2*. The retention time (min) depends on the differences in the heteroduplex composition: one elution curve represents the homoplasmic state (mutated or wild-type), two elution curves indicate heteroplasmy. Patient I.2: heteroplasmy in blood (N) and near-homoplasmic mutated in adenoma (T). Patient II.4: heteroplasmy in blood (N) and in normal parathyroid tissue (N*); near-homoplasmic mutated in the parathyroid carcinoma (T). Patient II.2: heteroplasmy in normal thyroid tissue (N*) and near-homoplasmic wild-type in the parathyroid carcinoma (T) and in the thyroid infiltration (TI). Patient II.3: near-homoplasmic mutated in both blood (N) and parathyroid adenoma (T). (**B**) Schematic representation of transcytoplasmatic hybrids (cybrids) generation. Osteosarcoma 143B.TK^−^ cells treated with ethidium bromide (BrEt) undergo mtDNA depletion resulting in Rho0 cell line generation. Rho0 cells are then fused with patients’ platelets, generating cybrids that carry various heteroplasmic loads of the mtDNA mutation (red: mitochondria carrying the m.2356A>G; blue: wild-type mt-DNA). (**C**) In gel activity for CI (CI-IGA) in cybrids carrying the homoplasmic m.2356A>G and wild-type mtDNA (WT). Two different CI-IGA experiments were carried out. For each experiment we have separated OXPHOS complexes from two independent mitochondrial protein extractions from pools of homoplasmic mutants (*n* = 2) and wild-type cells (*n* = 2). Band intensity was quantified by densitometry and data were normalized on untreated (UT) samples (mean ± SEM). (**D**) Heteroplasmic levels (%) of *MT-RNR2* mutations in I.2 adenoma. The fraction of *MT-RNR2* molecules carrying only the germline m.2356A>G is indicated in orange; the fraction of molecules carrying both the germline m.2356A>G and the somatic m.2635G>A is indicated in blue; the m.2635G>A has not been detected alone; wild-type (WT) *MT-RNR2* is indicated in green.

Among the mtDNA mutations detected, our attention was drawn onto the rare homoplasmic m.2356A>G mapping in the *MT-RNR2* gene that encodes the 16S mitochondrial ribosomal RNA (Table 1). This mutation was shared among II.4, I.2 and II.3, but absent from the tumor tissue of II.2, which prompted us to understand if it could be germline and transmitted from mother (I.2) to offspring, albeit with variable heteroplasmy. Indeed, the mutation was present in heteroplasmy in the mtDNA extracted from all the subjects’ peripheral blood (Figure 3A), including II.2.

We reasoned that if this mutation was counter-selected within the aggressive carcinoma of II.2, while shifting to homoplasmy in the other three less aggressive tumors, it might have a functional role (Figure 3A). To delve into the issue, we exploited the transcytoplasmatic hybrid (cybrid) technique that allows to transfer a mtDNA haplotype from a donor to a cell model in which the recipient mtDNA has been previously depleted (Figure 3B). Mitochondrial protein synthesis was evaluated through the analysis of CI re-assembly kinetics after the withdrawal of the mitochondrial protein synthesis inhibitor doxycycline. Interestingly, wild-type cybrids were able to promptly recover protein synthesis and reconstitute a fully assembled functional CI more rapidly compared to homoplasmic mutant cybrids, indicating that the m.2356A>G/*MT-RNR2* mutation induces an impairment of the mitoribosome activity (Figure 3C).

Overall, these data suggest the II.2 tumor, where the m.2356A>G was counter-selected, ought to have the most efficient mitochondrial protein synthesis of the four tumors analyzed, whereas the I.2 adenoma may likely suffer from a highly impaired mitochondrial translation, as it harbored two different pathogenic mutations on the same 16S ribosomal subunit. Indeed, cloning and sequencing of isolated mtDNA fragments spanning the *MT-RNR2* gene demonstrated that the somatic m.2635G>A mutation was on the same molecule as the germline m.2356G>A (Figure 3D), as the allele separation yielded 51% of clones carrying both mutations, 24.5% with only the m.2356A>G, and 24.5% harboring neither. No clones showed the m.2635G>A alone (Figure 3D), suggesting that the somatic mutation has a heteroplasmic load of about 50% and might have occurred relatively early in the tumor development on the same molecule of mtDNA carrying the germline mutation. This was suggestive of a synergistic effect possibly occurring between the two rare variants to generate a defective ribosome. 

### 3.4. IHC Staining for Respiratory Complexes Highlights a Correlation between Pathogenic mtDNA Mutations and Phenotype Severity

**Figure 4:**
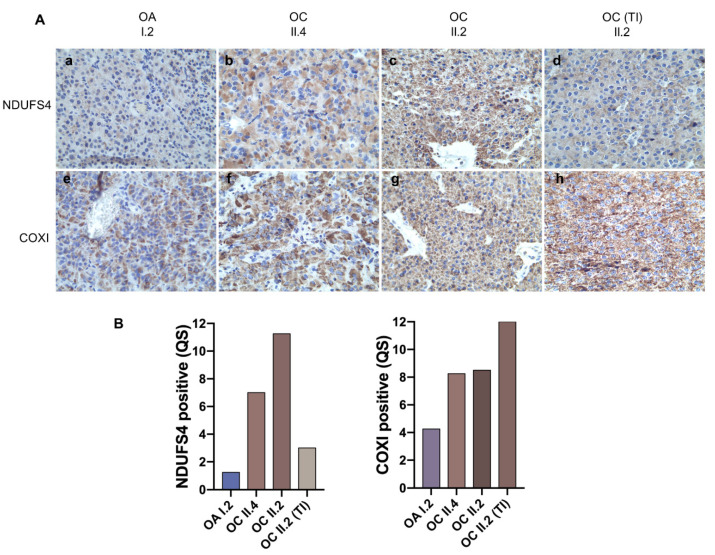
Correlation between pathogenic mtDNA mutations and phenotype severity. (**A**) Representative images are shown of the immunohistochemistry staining for CI subunit NDUFS4 (**a**–**d**) and CIV subunit COXI (**e**–**h**). Magnification 40×. (**B**) Quantification of NDUFS4 and COXI positive staining in HPT-JT tumors.

Once the full spectrum of mutations was acquired in all tumors, we sought evidence for a pathogenic effect that the combination of variants may have on respiratory complexes. Indeed, it is widely accepted that mtDNA mutations in oncocytic tumors often lead to disassembly of one or more OXPHOS complexes, which can be highlighted by validated IHC staining for specific protein subunits, such as nuclear-encoded NDUFS4 [13,46,54,55,56,57] or mtDNA-encoded CIV subunit COXI to infer the pathogenic potential of tRNA and rRNA mutations, whose effects may impinge on mitochondrial protein synthesis [58,59]. We hence exploited these validated methods on the family tumors for which sufficient material was available. NDUFS4 staining was faint in the I.2 oncocytic adenoma and predictive of a structural CI derangement, in agreement with its rather indolent clinical behavior, and in correlation with a mild COXI IHC positivity (Figure 4A(a,e)).

These findings were strongly suggestive of a synergistic role of the two rRNA mutations detected in this tumor (the germline m.2356A>G and the somatic m.2653A>G) in hampering mtDNA-encoded protein synthesis, such as ND subunits of CI, in turn affecting CI stability. A faint and heterogeneous NDUFS4 staining was also observed in the II.4 tumor along with a generally positive but heterogeneous COXI staining (Figure 4A(b,f)), suggesting the *MT-ND1* missense mutation m.3380G>A may have an effect on CI integrity, and that its mutation load reflects a population, not a cell, heteroplasmy. These data also suggest the germline m.2356A>G may play a role in reducing mtDNA-encoded protein synthesis, albeit not as strongly as in combination with a somatic rRNA mutation, as in I.2. Lastly, IHC in the most aggressive and infiltrating II.2 neoplasm displayed a strong and homogenous staining for both NDUFS4 and COXI (Figure 4A(c,g)), pointing to a functional and assembled respiratory chain within the hyperplastic mitochondria of this carcinoma. Interestingly, besides a strong positive staining for COXI in the thyroid infiltrate of the II.2 carcinoma, the latter presented a nearly negative NDUFS4 staining, in correlation with the CI *MT-ND6* mutation detected exclusively in this part of the tumor (Figure 4A(d,h)). Ultimately, taking together the identification of mtDNA variants and the establishment of their pathogenicity, with regard on their effect on CI integrity, HPT-JT tumors showed an inverse correlation between the load of pathogenic mtDNA mutations found in each tumor and phenotype severity.

## 4. Discussion

It is nowadays widely accepted that solid cancers require an intact respiratory chain to thrive, despite relying preferentially on a glycolytic metabolism. It has been postulated that this is due to the need to carry out an efficient anaplerosis, whereby even when glutamine is used as a carbon source alternatively to glucose, both the Krebs cycle enzymes and the OXPHOS system must be functional to warrant anabolism. In agreement with this notion, highly pathogenic mtDNA mutations have been shown to be counter-selected in cancers [9] with the notable exception of oncocytoma, where they often determine a disassembly of respiratory complexes, mainly CI, instead [60,61]. Occurrence of such disruptive mutations in sporadic oncocytic tumors has been rarely associated with a nuclear oncogenic driver, with scarce reports in the literature [62,63], whereas in familial forms of oncocytoma, where the driver is known, mtDNA mutations either seem to be lacking [64] or occur in the germline and become favorably selected in the neoplasm [65]. Whenever disassembling mtDNA mutations trespass a threshold for a phenotypic effect, however, cancer cells acquire an indolent, low-replicating and low-aggressive profile, likely due to a slowdown of their metabolic activity [11,66], in a tight resemblance with an aging phenotype, where mutations in mtDNA become fixed and may contribute to tissue senescence [67].

We report here for the first time the occurrence of oncocytic transformation in several tumors in a family pedigree, associated with HPT-JT syndrome, an inherited cancer-predisposing disorder caused by germline mutations in the *CDC73* tumor suppressor gene. In contrast with previous rare familial oncocytic cancers, however, different somatic and pathogenic mtDNA mutations were shown to accumulate on the genetic background of a pre-existing germline rare variant in all affected members, transmitted at high heteroplasmy levels from mother to offspring. We provided a functional demonstration of the impact that the inherited rRNA mutation may have on mitochondrial translation efficiency, which we expect to be relevant in tissues with high metabolic activity such as cancer. Indeed, in a parallel fashion to neuromuscular diseases [68], even heteroplasmic low-penetrance mutations mapping on mitochondrial translation genes such as tRNAs and rRNAs may display deleterious consequences in tissues with high energy requirements and mitochondrial turnover [69,70]. We also show that, for some of the somatic mtDNA mutations occurring in the oncocytic neoplasms, their pathogenicity is reflected in the abundance of respiratory complexes as evaluated via validated IHC markers [13,46,54,55,56]. It is striking that an increasing gradient of positive OXPHOS staining correlates with both a decreasing number of pathogenic mtDNA mutations and more evident malignant features (including the mitotic Ki67 index), with the most indolent adenoma harboring the highest number of pathogenic mutations and the most aggressive and fatal carcinoma purifying the pathogenic germline variant. Our findings, therefore, support the view that an intact and potentially functional OXPHOS chain contributes to confer malignancy and aggressive features, such as in the case of the II.2 infiltrating carcinoma. These data also point to the usefulness of respiratory complexes staining, or mtDNA sequencing, as prognostic tools that may help predict clinical behavior by suggesting the metabolic capability of neoplasms.

We do not know whether the occurrence of *CDC73* driver mutations may play a role in oncocytic transformation, as apparently it has no direct mitochondrial functions. The gene-encoded protein, parafibromin, plays a role in chromatin remodeling [71] and is part of a transcriptional complex in which polymerase II binds to drive mRNA synthesis [72]; it is therefore plausible and tempting to speculate that a dysfunctional *CDC73* may affect transcription of nuclear-encoded mitochondrial genes too, leading to the organelle derangement. It is worth mentioning that *CDC73* somatic mutations have been detected in a small number of sporadic renal oncocytomas in one study [73], which however does not appear sufficient to infer a causative role of parafibromin in oncocytic transformation *per se*. While this warrants further investigation, the germline rRNA mtDNA mutation may be the genetic determinant responsible to generate the underlying conditions predisposing to the onset of an oncocytic phenotype. The latter has been postulated to be secondary to cell transformation [51,63], and the relaxed selection of somatic mtDNA mutations ought to occur in a permissive microenvironment. When mitochondria are inefficient, such as they may be in the presence of the m.2356A>G mutation, and not used in favor of glycolysis as during the Warburg effect in cancer cells [74], it is plausible that mtDNA mutations are free to accumulate over the phenotypic threshold, triggering a short-circuited compensatory mitochondrial biogenesis and hence oncocytic transformation [75]. As OXPHOS function must be restored by cancer cells during late stages of tumor progression [6] to sustain anaplerosis and anabolism in general, only tumors that become rid of pathogenic mtDNA mutations may proceed to malignancy, as in the infiltrating carcinoma we described. It would be most interesting to follow over time the clonal expansion of cancer cells carrying the heteroplasmic m.14387A>G/*MT-ND6*, acquired within the infiltration of II.2 and absent in the rest of the mass, as the weak staining of NDUFS3 only within the infiltration is suggestive of a reduced CI integrity: whether cancer cells keep accumulating or eliminate the heteroplasmic variant to recover a fully functional OXPHOS would likely make the difference between a regression to indolence or malignant progression.

## 5. Conclusions

In conclusion, investigating familial forms of oncocytic tumors allows to infer the dynamic and plastic shaping of the mitochondrial genome within cancer cells, where it contributes to orienting the energetic capabilities and overall metabolic performance that may drive a tumor towards malignancy, or to devolve into an indolent entity.

## Figures and Tables

**Table 1:** MtDNA mutations found in the HPT-JT family. AA: amino acid; AF: allele frequency (from HmtVar); DS: disease score (from HmtVar); OA: oncocytic adenoma; OC: oncocytic carcinoma; VUS: variant of uncertain significance.

Family ID	Base Change	AA Change	Mutation Status	Locus	AF Healthy/Patients	DS	Pathogenicity Prediction
I.2 OA	m.2356A>G	-	Germline	MT-RNR2	0.00026/0.00021	ND	Pathogenic *
	m.2635G>A	-	Somatic	MT-RNR2	-	-	VUS
	m.14973G>A	G76D	Somatic	MT-CYB	0.00000/0.00000	0.91	Pathogenic
II.4 OC	m.2356A>G *	-	Germline	MT-RNR2	0.00026/0.00021	ND	Pathogenic *
	m.5147G>A	silent	Somatic	MT-ND2	0.04370/0.04061	ND	Benign
	m.3380G>A	R25Q	Somatic	MT-ND1	0.00005/0.00043	0.88	Pathogenic
II.2 OC	m.14387A>G	L96S	Somatic	MT-ND6	0.00000/0.00000	0.74	Pathogenic
II.3 OA	m.2356A>G *	-	Germline	MT-RNR2	0.00026/0.00021	ND	Pathogenic *
	m.10371G>A	E105K	Somatic	MT-ND3	0.00000/0.00022	0.89	Pathogenic

* Pathogenicity assessed here for the first time.

## Supplementary Materials

The following are available online at https://www.mdpi.com/article/10.3390/cells10112920/s1, Supplementary Table S1: mtDNA variants annotation, Supplementary Figure S1: Haematoxylin and eosin staining of II.3 oncocytic adenoma.
