# Supplementary material for: Pathogenic Mitochondrial DNA Mutation Load Inversely Correlates with Malignant Features in Familial Oncocytic Parathyroid Tumors Associated with Hyperparathyroidism-Jaw Tumor Syndrome
Source: Cells. 2021 Oct 28;10(11):2920. doi: 10.3390/cells10112920 (PMC8616364; doi:10.3390/cells10112920)
Supplement: Supplementary file 1 [file cells-10-02920-s001.zip › Supplementary files/Supplementary figure 1.pdf]

Supplementary figure 1

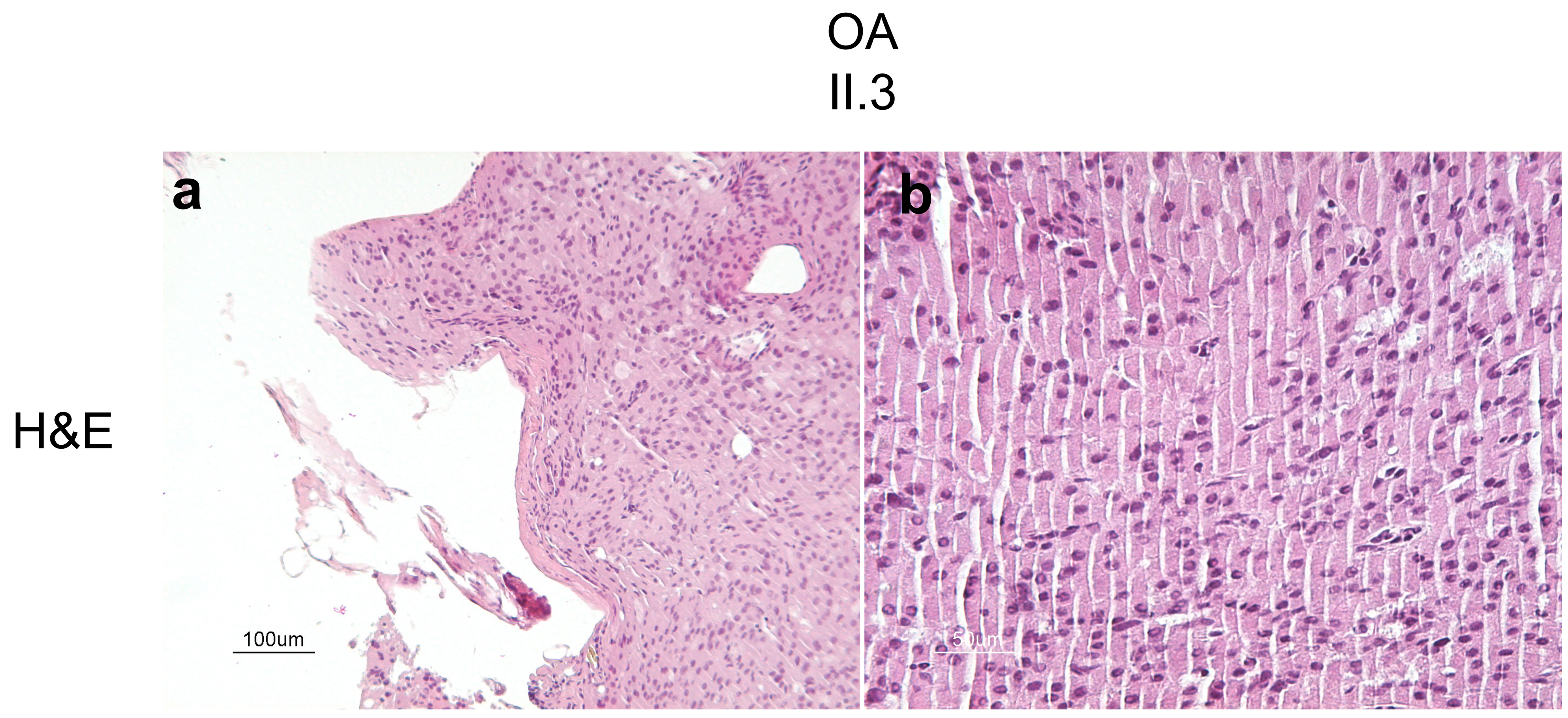

Figure 1. Hematoxylin and eosin (H&E) staining. OA - Parathyroid oncocytic adenoma, magnification: 10x in a and 20x in b.
